# Non-Mydriatic Fundus Retinography in Screening for Diabetic Retinopathy: Agreement Between Family Physicians, General Ophthalmologists, and a Retinal Specialist

**DOI:** 10.3389/fendo.2018.00251

**Published:** 2018-05-18

**Authors:** Leonardo Provetti Cunha, Evelyn Alvernaz Figueiredo, Henrique Pereira Araújo, Luciana Virgínia Ferreira Costa-Cunha, Carolina Ferreira Costa, José de Melo Costa Neto, Aline Mota Freitas Matos, Marise Machado de Oliveira, Marcus Gomes Bastos, Mário Luiz Ribeiro Monteiro

**Affiliations:** ^1^Department of Ophthalmology, School of Medicine, Federal University of Juiz de Fora, Juiz de Fora, Brazil; ^2^Juiz de Fora Eye Hospital, Juiz de Fora, Brazil; ^3^Division of Ophthalmology, University of São Paulo Medical School, São Paulo, Brazil; ^4^Division of Family Medicine, School of Medicine, Federal University of Juiz de Fora, Juiz de Fora, Brazil; ^5^Department of Nephrology, School of Medicine, Federal University of Juiz de Fora, Juiz de Fora, Brazil

**Keywords:** diabetic retinopathy, macular edema, non-mydriatic fundus retinography, telemedicine, diabetes mellitus, family physicians, visual loss

## Abstract

**Purpose:**

To determine the level of agreement between trained family physicians (FPs), general ophthalmologists (GOs), and a retinal specialist (RS) in the assessment of non-mydriatic fundus retinography in screening for diabetic retinopathy (DR) in the primary health-care setting.

**Methods:**

200 Diabetic patients were submitted to two-field non-mydriatic digital fundus camera. The images were examined by four trained FPs, two GOs, and one RS with regard to the diagnosis and severity of DR and the diagnosis of macular edema. The RS served as gold standard. Reliability and accuracy were determined with the kappa test and diagnostic measures.

**Results:**

A total of 397 eyes of 200 patients were included. The mean age was 55.1 (±11.7) years, and 182 (91%) had type 2 diabetes. The mean levels of serum glucose and glycosylated hemoglobin A1c were 195.6 (±87.3) mg/dL and 8.9% (±2.1), respectively. DR was diagnosed in 166 eyes by the RS and in 114 and 182 eyes by GO_1_ and GO_2_, respectively. For severity, DR was graded as proliferative in 8 eyes by the RS vs. 15 and 9 eyes by GO_1_ and GO_2_, respectively. The agreement between the RS and the GOs was substantial for both DR diagnosis (GO_1_ *k* = 0.65; GO_2_ *k* = 0.74) and severity (GO_1_ *k* = 0.60; GO_2_ *k* = 0.71), and fair or moderate for macular edema (GO_1_ *k* = 0.27; GO_2_ *k* = 0.43). FP_1_, FP_2_, FP_3_, and FP_4_ diagnosed DR in 108, 119, 163, and 117 eyes, respectively. The agreement between the RS and the FPs with regard to DR diagnosis was substantial (FP_2_ *k* = 0.69; FP_3_ *k* = 0.73; FP_4_ *k* = 0.71) or moderate (FP_1_ *k* = 0.56). As for DR severity, the agreement between the FPs and the RS was substantial (FP_2_ *k* = 0.66; FP_3_ *k* = 069; FP_4_ *k* = 0.64) or moderate (FP_1_ *k* = 0.51). Agreement between the FPs and the RS with regard to macular edema was fair (FP_1_ *k* = 0.33; FP_2_ *k* = 0.39; FP_3_ *k* = 0.37) or moderate (FP_4_ *k* = 0.51).

**Conclusion:**

Non-mydriatic fundus retinography was shown to be useful in DR screening in the primary health-care setting. FPs made assessments with good levels of agreement with an RS. Non-mydriatic fundus retinography associated with appropriate general physicians training is essential for the DR screening.

## Introduction

Diabetic retinopathy (DR) is one of the main complications of diabetes mellitus (DM) and the main cause of preventable blindness in the world, especially in economically active populations in developed countries, affecting and threatening the vision of over 12.6 million and 37.3 million people, respectively (1, 2). In many countries, a large proportion of public health-care funds are destined for the treatment, rehabilitation, and social security expenditures of persons with DR (3–5), making public policies for the prevention of DR-related blindness more imperative than ever.

Screening for DR is crucial due to the frequent absence of symptoms, even in advanced stages, and investments in prevention have proven cost-effective (5, 6). The incidence of DR is very high and growing. However, though desirable, ophthalmological evaluation of every single diabetic individual is unfeasible from a practical and economic point of view, especially if no priorities are defined.

In Brazil, a minor part of the population is covered by private health insurance. Primary health care is provided at public health facilities manned by multidisciplinary teams, which include a family physician. Following the first examination, patients requiring specialized care may be referred to secondary-level medical services. Ideally, screening for diseases such as DR should be performed at primary health-care facilities. This can be done by direct ophthalmoscopy, a relatively inexpensive and accessible method. However, as shown by ample evidence, this important tool is underused in Brazilian primary health care (7). Another attractive option for DR screening is non-mydriatic fundus retinography, a diagnostic device that provides detailed images of the eye fundus and obtain high-quality images of the retina and optic nerve head, covering a total area of 45°. Despite the somewhat high initial cost of the equipment, obtaining retinal images with the device is simple, fast, inexpensive, and requires no pupil dilation. Furthermore, the fundus images can be acquired by trained non-physicians and storaged for evaluation by an ophthalmologist, if necessary (8–13).

The purpose of this study was to determine the level of agreement between trained family physicians (FPs), general ophthalmologists (GOs), and a retinal specialist (RS) in the assessment of non-mydriatic fundus retinography in DR screening in the primary health-care setting.

## Materials and Methods

In this cross-sectional study, diabetic patients were recruited at a referral service (IMEPEN Foundation) in Juiz de Fora (a city in Minas Gerais, Brazil), between February and July 2016. The study protocol followed the principles of the Declaration of Helsinki and was approved by the Research Ethics Committee of the Federal University of Juiz de Fora, Minas Gerais, Brazil (protocol number 43368415.1.0000.5147). All participants gave their informed written consent.

**Figure 1:**
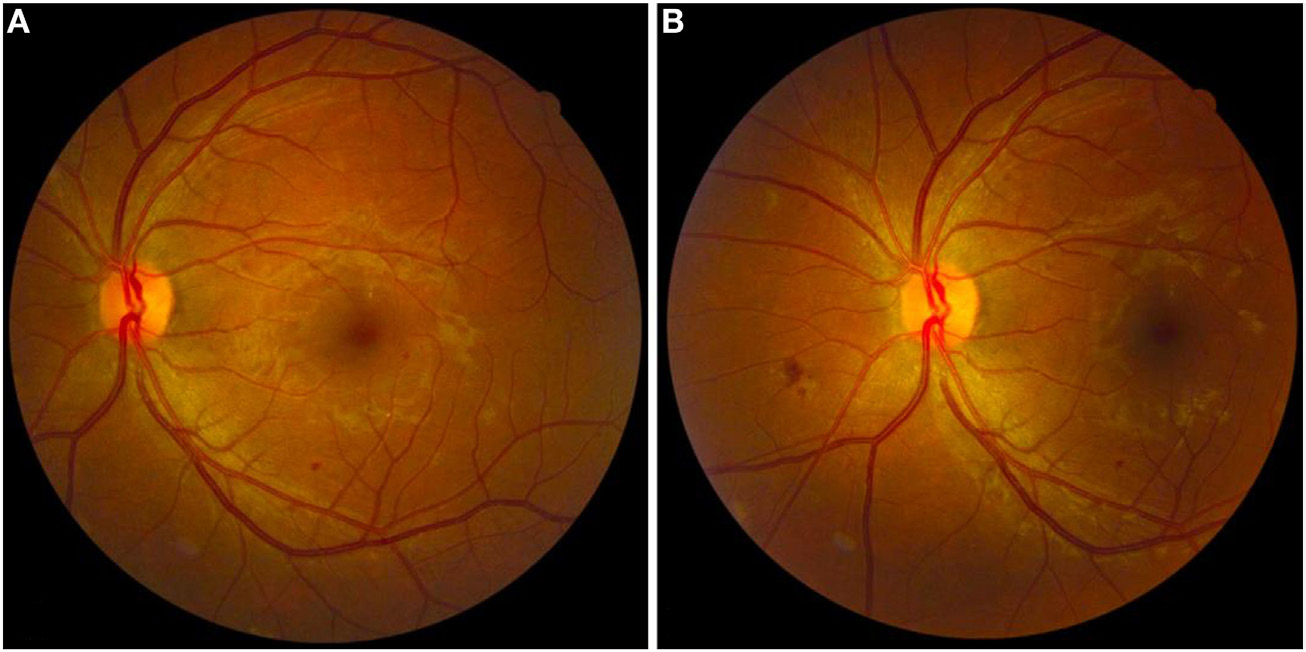
Fundus retinal images of the left eye of a patient with diabetic retinopathy. **(A)** Fundus retinography centered on the fovea. **(B)** Fundus retinography centered on the optic disk. Note the presence of small hard exudate, microaneurysms, and dot-blot retinal hemorrhages around macular area **(A)** and small cotton wool spots and superficial retinal hemorrhages in the nasal area of the optic disk **(B)**. The pictured eye was graded as “non-proliferative” by the retinal specialist.

A total of 794 fundus images from 397 eyes of 200 patients diagnosed with DM were produced with a 45° non-mydriatic retinal camera (Canon CR-2, Canon, Tokyo, Japan) and analyzed. Two medical students were trained by medical supervisors in how to operate the non-mydriatic fundus camera and acquire images. The patient was seated in a darkened room and both eyes were photographed using a two-field protocol: the first centered on the fovea, the second on the optic disk (Figure 1). The pupils were not dilated.

Inclusion criteria was as follows: patients with diagnosis of type 1 or type 2 DM; age range between 18 and 70 years old; good general healthy condition; being able to collaborate with the examination and agreement to participate in the study. Exclusion criteria were as follows: patients under 18 or older than 70 years old; physical and mental conditions that made acquisition of fundus examination impossible; media opacities that prevented the acquisition of fundus images and patients who refused to participate to the study.

The stored digital images were graded by four FPs, two GOs, and one RS at random. The diagnosis was based on two photos of each eye (two-field protocol) to avoid discrepancies due to the possible absence of retinopathy in one of the images. All graders used a 19″ flat computer screen to view the images. Provided and certified by an RS, training in image analysis consisted of two 3-h sessions per week during 3 months. For each set of images, the graders verified the presence/absence of DR, the severity of the condition (absent, non-proliferative, proliferative, treated, and non-gradable), and the presence/absence of macular edema. DR was diagnosed based on the presence of one or more of the following findings: microaneurysms, hard exudate, cotton wool spots, and retinal/optic disk neovascularization. Severity was graded as follows: absent, non-proliferative (one or more of the above findings, except neovascularization), proliferative (neovascularization, with or without the findings above, and with or without tractional retinal detachment and preretinal hemorrhage), treated DR (chorioretinal scars caused by argon retinal laser photocoagulation) (14), and non-gradable (ambiguous findings or poor image quality). Macular edema was defined as retinal edema, retinal thickening or hard exudates within 500 µm of the center of the fovea, and retinal edema or thickening of one disk diameter or larger in any location, with any part within one disk diameter of the center of the macula (15, 16). Clinical information was also collected for each patient. Quantitative data were converted into abstract measures (mean, SD, median, minimum, and maximum) while qualitative data were expressed as absolute and relative frequencies. The graders were blinded to the patient data.

The presence/absence and severity of DR and the presence/absence of macular edema were expressed in absolute and relative frequencies, and the agreement between the RS (main examiner, gold standard) and the FPs and GOs was evaluated using the kappa test and diagnostic measures (sensitivity, specificity, positive predictive value, and negative predictive value). Agreement was scored as poor (0–0.19), fair (0.2–0.39), moderate (0.4–0.59), substantial (0.6–0.79), or almost perfect (0.8–1.00) (17). The total agreement between ophthalmologists and non-ophthalmologists was calculated using the respective coefficients of agreement. All statistical analyses were performed with the software IBM-SPSS for Windows v. 20.0. The level of statistical significance was set at 5% (*p* < 0.05).

## Results

A total of 397 eyes of 200 patients were included in the analysis. Three eyes (corresponding to 6 fundus retinographies) were excluded due to advanced media opacity or insufficient pupil size. The mean age was 55.1 (±11.7) years, 122 (61%) patients were female, and 182 (91%) had type 2 diabetes. The mean disease duration was 130.2 (±97.8) months. The mean systolic and diastolic arterial pressure was 129.7 (±15.3) and 81.9 (±8.9) mmHg, respectively. The mean serum glucose level was 195.6 (±87.3) mg/dL, the mean glycosylated hemoglobin A1c (HbGA1C) rate was 8.9% (±2.1), and the mean serum creatinine level was 0.88 (±0.3) mg/dL. Table 1 contains a summary of the clinical findings.

**Table 1:** Demographic and clinical data in 200 diabetic patients included in the study.

Parameter	Description
**Age (years)**	
Mean ± SD	55.1 ± 11.7
Median (min; max)	56 (19; 76)
**Sex,*n*(%)**	
Male	78 (39%)
Female	122 (61%)
**Disease duration (months)**	
Mean ± SD	130.2 ± 97.8
Median (min; max)	120 (12; 444)
**Type of diabetes,*n*(%)**	
Type 1	18 (9%)
Type 2	182 (91%)
**Systolic blood pressure**	
Mean ± SD	129.7 ± 15.3
Median (min; max)	120 (100; 190)
**Diastolic blood pressure**	
Mean ± SD	81.9 ± 8.9
Median (min; max)	80 (60; 130)
**Fasting glucose level**	
Mean ± SD	195.6 ± 87.3
Median (min; max)	179.5 (50; 550)
**Glycated hemoglobin level**	
Mean ± SD	8.9 ± 2.1
Median (min; max)	8.44 (4.6; 17)
**Creatinine level**	
Mean ± SD	0.88 ± 0.3
Median (min; max)	0.83 (0.4; 2.3)

Diabetic retinopathy was diagnosed in 166 (41.8%) eyes by the RS and in 114 (28.7%) and 182 (45.8%) eyes by GO_1_ and GO_2_, respectively. The overall agreement between the RS and the GOs with regard to DR diagnosis was substantial (*k* = 0.65 and 0.74 for GO_1_ and GO_2_, respectively). As for severity, DR was graded as proliferative in 8 eyes (2%) by the RS vs. 15 eyes (3.8%) and 9 eyes (2.3%) by GO_1_ and GO_2_, respectively, and as non-proliferative in 143 eyes (36%) by the RS vs. 82 eyes (20.7%) and 155 eyes (39%) by GO_1_ and GO_2_, respectively. The overall agreement between the RS and the GOs with regard to severity was also substantial (*k* = 0.60 and 0.71 for GO_1_ and GO_2_, respectively). Finally, the RS detected macular edema in 88 (22.2%) eyes, whereas GO_1_ and GO_2_ detected it in 20 eyes (5%) and 30 eyes (7.6%), respectively, indicating a fair (GO_1_ *k* = 0.27) or moderate (GO_2_ *k* = 0.43) agreement between the groups (Table 2).

**Table 2:** Diagnostic analysis of non-mydriatic fundus images from diabetic patients and inter-grader agreement between a retinal specialist (RS) and two general ophthalmologists (GO).

Variable	RS	GO_1_	GO_2_
**Diabetic retinopathy**			
Absent (%)	231 (58.2)	283 (71.3)	215 (54.2)
Present (%)	166 (41.8)	114 (28.7)	182 (45.8)
Kappa (95% CI)		0.65 (0.57; 0.721)	0.74 (0.67; 0.81)
Sensitivity (95% CI)		64.8 (57; 72.1)	89.7 (84; 93.9)
Specificity (95% CI)		97 (93.9; 98.8)	85.3 (80.1; 89.6)
PPV (95% CI)		93.9 (87.8; 97.5)	81.3 (74.9; 86.7)
PNV (95% CI)		79.5 (74.3; 84.1)	92.1 (87.6; 95.3)
**Severity**			
Absent (%)	231 (58.2)	283 (71.3)	215 (54.2)
Treated (%)	15 (3.8)	17 (4.3)	17 (4.3)
Non-proliferative (%)	143 (36)	82 (20.7)	155 (39)
Proliferative (%)	8 (2)	15 (3.8)	9 (2.3)
Non-gradable (%)	0 (0)	0 (0)	1 (0)
Weighted kappa (95% CI)		0.60 (0.53; 0.68)	0.71 (0.64; 0.77)
**Macular edema**			
Absent (%)	309 (77.8)	377 (95)	367 (92.4)
Present (%)	88 (22.2)	20 (5)	30 (7.6)
Kappa (95% CI)		0.27 (0.17; 0.38)	0.43 (0.32; 0.54)
Sensitivity (95% CI)		20.5 (12.6; 30.4)	33 (23.3; 43.8)
Specificity (95% CI)		99.4 (97.7; 99.9)	99.7 (98.2; 100)
PPV (95% CI)		90 (68.3; 98.8)	96.7 (82.8; 99.9)
PNV (95% CI)		81.4 (77.1; 85.2)	83.9 (79.8; 87.5)

*CI, confidence interval; PPV, predictive positive value; PNV, predictive negative value*.

FP_1_, FP_2_, and FP_4_ diagnosed DR in roughly the same number of eyes (108, 119, and 117, respectively), while FP_3_ came closer to the RS (166 vs. 163, respectively). Thus, agreement between the FPs and the RS with regard to DR diagnosis was good for FP_2_, FP_3_, and FP_4_ (*k* = 0.69, 0.73 and 0.71, respectively) and moderate for FP_1_ (*k* = 0.56). As for DR severity, the overall agreement between the FPs and the RS was substantial (*k* = 0.66, 0.69, and 0.64 for FP_2_, FP_3_, and FP_4_, respectively) or moderate (*k* = 0.51; FP_1_). Agreement between the FPs and the RS with regard to macular edema was fair (*k* = 0.33, 0.39, and 0.37 for FP_1_, FP_2_, and FP_3_, respectively) or moderate (*k* = 0.51 for FP_4_) (Table 3). The number of non-gradable eyes was small in all groups (Tables 2 and 3).

**Table 3:** Diagnostic analysis of non-mydriatic fundus images from diabetic patients and inter-grader agreement between a retinal specialist (RS) and four trained family physicians (FPs).

Variable	RS	FP_1_	FP_2_	FP_3_	FP_4_
**Diabetic retinopathy**					
Absent (%)	231 (58.2)	289 (72.8)	278 (70)	234 (58.9)	280 (70.5)
Present (%)	166 (41.8)	108 (27.2)	119 (30)	163 (41.1)	117 (29.5)
Kappa (95% CI)		0.56 (0.48; 0.64)	0.69 (0.61; 0.76)	0.73 (0.66; 0.80)	0.71 (0.64; 0.78)
Sensitivity (95% CI)		58.2 (50.3; 65.8)	68.5 (60.8; 75.5)	83.6 (77.1; 88.9)	69.1 (61.4; 76)
Specificity (95% CI)		94.8 (91.1; 97.3)	97.4 (94.5; 99)	89.2 (84.5; 92.9)	98.7 (96.3; 99.7)
PPV (95% CI)		88.9 (81.4; 94.1)	95 (89.3; 98.1)	84.7 (78.2; 89.8)	97.4 (92.7; 99.5)
PNV (95% CI)		76.1 (70.8; 80.9)	81.3 (76.2; 85.7)	88.5 (83.7; 92.3)	81.8 (76.8; 86.1)
**Severity**					
Absent (%)	231 (58.2)	289 (72.8)	278 (70)	234 (58.9)	280 (70.5)
Treated (%)	15 (3.8)	15 (3.8)	14 (3.5)	20 (5)	14 (3.5)
Non-proliferative (%)	143 (36)	72 (18.1)	100 (25.2)	132 (33.2)	66 (16.6)
Proliferative (%)	8 (2)	21 (5.3)	5 (1.3)	9 (2.3)	37 (9.3)
Non-gradable (%)	0 (0)	0 (0)	0 (0)	2 (0.5)	0 (0.0)
Weighted kappa (95% CI)		0.51 (0.44; 0.59)	0.66 (0.59; 0.73)	0.69 (0.62; 0.76)	0.64 (0.58; 0.71)
**Macular edema**					
Absent (%)	309 (77.8)	363 (91.4)	362 (91.2)	358 (90.2)	343 (86.4)
Present (%)	88 (22.2)	34 (8.6)	35 (8.8)	39 (9.8)	54 (13.6)
Kappa (95% CI)		0.33 (0.21; 0.44)	0.39 (0.28; 0.51)	0.37 (0.26; 0.48)	0.51 (0.40; 0.62)
Sensitivity (95% CI)		28.4 (19.3; 39)	33 (23.3; 43.8)	33 (23.3; 43.8)	47.7 (37; 58.6)
Specificity (95% CI)		97.1 (94.5; 98.7)	98.1 (95.8; 99.3)	96.8 (94.1; 98.4)	96.1 (93.3; 98)
PPV (IC 95%)		73.5 (55.6; 87.1)	82.9 (66.4; 93.4)	74.4 (57.9; 87)	77.8 (64.4; 88)
PNV (IC 95%)		82.6 (78.3; 86.4)	83.7 (79.5; 87.4)	83.5 (79.3; 87.2)	86.6 (82.5; 90)

*CI, confidence interval; PPV, predictive positive value; PNV, predictive negative value*.

## Discussion

The overall prevalence of diagnosed DR (e.g., 41.8% for the RS) was higher in this study than in most of the reviewed literature. Thus, when evaluating retinal photographs of 153 consecutive diabetic patients at a referral service, Siu et al. found a prevalence of 15% (18). Using a non-mydriatic fundus camera, Bhargava et al. found a prevalence of 17.8% in a sample of 367 diabetic patients from two primary health-care clinics in Singapore (19). More recently, 9,347 patients with type 1 and 2 DM studied by Vujosevic et al. yielded an overall prevalence of 27.6% (20). Our findings may in part be explained by sampling bias: our patients came from the largest public DM referral service in Juiz de Fora, where severe cases are usually treated. This is supported by the long mean disease duration (130.2 months) and the high mean levels of serum glucose (195.6 mg/dL) and HbA1c (8.9%) in our sample.

As method of DR screening, direct ophthalmoscopy can be difficult to perform and requires frequent retraining. Moreover, it only allows to visualize a small area of the fundus, increasing the risk of false-negative results and misdiagnosis (7). In Siu et al., non-mydriatic fundus retinography was found by ophthalmologists to be more sensitive than direct ophthalmoscopy (64 vs. 41%) in the detection of DR in a sample of diabetic patients (18). Likewise, Taylor et al. concluded that non-mydriatic retinal photography was at least as efficient as direct mydriatic ophthalmoscopy at screening for DR, and better at detecting exudative maculopathy (21).

Our results suggest non-mydriatic fundus retinography is a useful and relatively reliable method of DR screening, which may be performed by trained nurses or technicians. Non-invasive, it avoids visual discomfort due to pharmacological mydriasis and the risk of triggering acute angle-closure glaucoma in predisposed patients. Moreover, it allows for storage and remote analysis of digital fundus images by RSs (telemedicine), increasing the availability of DR screening for socioeconomically challenged patients and rural populations (22–24).

Another important concern is the ideal number of fundus images for proper screening. Seven-field fundus retinography was previously considered the gold standard for DR screening, but acquisition can be time-consuming and costly, limiting the usefulness of the protocol (18, 25). Several authors have shown that two 45° fundus images (two-field protocol) are more sensitive than single fundus images and sufficient for reliable DR screening (26–28). More recently, high levels of sensitivity have been reported for non-mydriatic ultrawide field retinal imaging. This promising technique broadens the view of the retina from 45° to 200° in a single image, increasing chances of identifying DR and peripheral lesions present in almost 10% of severe cases (29–31).

The main purpose of our study was to evaluate the level of agreement between FPs, GOs, and an RS in the assessment of non-mydriatic fundus images of diabetic patients. The inclusion of FPs in the study design is justified by the fact that, in Brazil, FPs are responsible for the provision of primary care. Thus, DM patients are usually controlled, followed, and referred for ophthalmological evaluation by FPs. Based on our findings, we recommend non-mydriatic fundus retinography as a complementary work-up for diabetic patients, helping in the diagnosis of DR and the setting of priorities for ophthalmic evaluations, making access to ophthalmologic care easier for severe patients and avoiding unnecessary testing of patients without DR. We believe the approach would have a substantial impact, not only on DR diagnosis, but on the prevention of visual loss in diabetic patients.

The agreement between the RS and the FPs was almost as good as that between the SR and the GOs (moderate to substantial with regard to both diagnosis and severity). As for the diagnosis of macular edema, the agreement between the RS and the FPs or GOs was fair to moderate, suggesting the latter two groups had similar diagnostic skills. In fact, the PFs performed better than expected, considering the short time of training. However, we believe that one of the most important concerns that should be emphasized in our study is a proper training of FPs in the assessment of the fundus images obtained by non-mydriatic fundus retinography of diabetic patients. Longer and more frequent training and periodic accreditations would further improve their diagnostic sensitivity and accuracy. In a recent study, Rosses et al. found high sensitivity (82.9%), specificity (92%) and accuracy (90.3%) for trained FPs evaluating diabetic patients for DR, and a substantial level of agreement (kappa-adjusted coefficient: 0.74–0.80) with GOs (32). Using a non-mydriatic fundus camera to assess 2,779 diabetic patients, Romero et al. (33) found a substantial level of agreement between FPs and GOs (kappa coefficient: 0.82), with high sensitivity (95.2%) and specificity (98%) for DR diagnosis, concluding that the inclusion of FPs in DR screening programs is feasible.

One of the limitations of our study is the absence of fundus examination (e.g., by slit lamp biomicroscopy or indirect binocular ophthalmoscopy) for comparison with fundus retinography, making it impossible to evaluate the diagnostic power of non-mydriatic retinography. Moreover, the effect of mydriasis on the diagnostic sensitivity of DR screening was not evaluated. Some authors favor the use of mydriasis to improve diagnostic performance (27, 32), but in view of the risk of serious complications (for example, acute angle-closure glaucoma), especially in the absence of an ophthalmologist, the improvement potentially afforded by mydriasis does not seem worthwhile. Despite some limitations, we believe it is the first study that makes a direct assessment of the agreement between FPs, GOs, and RS in a DR screening using non-mydriatic fundus camera. Our results draw attention to the fact that in diabetic patients, evaluation of fundus image by trained FPs can be effective with results comparable with those of GOs, optimizing resources by selecting patients who do need further specialized retinal evaluation and treatment.

In conclusion, our study shows that two-field non-mydriatic fundus retinography is a useful tool in DR screening in the primary health-care setting and that trained FPs can make DR assessments with adequate levels of sensitivity and specificity and substantial levels of agreement with an RS with regard to both diagnosis and grading. We believe that the enabling, through public policies, of primary care physicians in Brazil (and perhaps elsewhere) to perform DR screening with non-mydriatic fundus retinography would significantly reduce the incidence of DR-related blindness.

## Ethics Statement

This study was carried out in accordance with the recommendations of the Declaration of Helsinki and was approved by the Institutional Review Board with written informed consent from all subjects. All subjects gave written informed consent in accordance with the Declaration of Helsinki. The protocol was approved by the Research Ethics Committee of the School of Medicine of the Federal University of Juiz de Fora.

## Author Contributions

Drafted the paper: LC and MM. Revised the paper: LC, EF, HA, LC-C, CC, JN, AM, MO, MB, and MM. Contributed with the design of the work: LC and MB. Contributed with the acquisition of the data: LC, EF, HA, LC-C, CC, JN, AM, MO, and MB. Contributed with analysis and interpretation of the data: LC, EF, HA, LC-C, CC, JN, AM, MO, and MM. Approved the final version of the manuscript: LC, EF, HA, LC-C, CC, JN, AM, MO, MB, and MM.

## Conflict of Interest Statement

The authors declare that the research was conducted in the absence of any commercial or financial relationships that could be construed as a potential conflict of interest.

## Abbreviations

FPs, family physicians; GOs, general ophthalmologists; RS, retinal specialist; DR, diabetic retinopathy; DM, diabetes mellitus; k, kappa test; SBP, systolic blood pressure; DBP, diastolic blood pressure; CI, confidence interval; PPV, predictive positive value; PNV, predictive negative value.
